# Characterization of RNA Editome in the Mammary Gland of Yaks during the Lactation and Dry Periods

**DOI:** 10.3390/ani12020207

**Published:** 2022-01-16

**Authors:** Xiaoyun Wu, Wondossen Ayalew, Min Chu, Jie Pei, Chunnian Liang, Pengjia Bao, Xian Guo, Ping Yan

**Affiliations:** 1 Key Laboratory of Yak Breeding Engineering, Lanzhou Institute of Husbandry and Pharmaceutical Sciences, Chinese Academy of Agricultural Sciences, Lanzhou 730050, China; wuxiaoyun@caas.cn (X.W.); wondessenayalew9@gmail.com (W.A.); chumin@caas.cn (M.C.); peijie@caas.cn (J.P.); chunnian2006@163.com (C.L.); baopengjia@caas.cn (P.B.); 2 Department of Animal Production and Technology, Wolkite University, Wolkite P.O. Box 07, Ethiopia

**Keywords:** RNA editing, mammary gland, lactation, yak, RNA-seq

## Abstract

**Simple Summary:**

In order to study the influence of RNA editing sites on lactation and mammary gland development process in yaks, we comprehensively characterized the RNA editome of the yak mammary gland during the lactation period and dry period by using the transcriptome and genome sequencing data. The results revealed 82,872 nonredundant RNA editing sites, 14,159 of which were differentially edited between the lactation period and dry period. Enrichment analysis showed that the genes harboring differential editing sites were mainly associated with mammary gland development-related pathways, such as MAPK pathway, PI3K-Akt pathway, FoxO signaling pathway, GnRH signaling pathway, and focal adhesion pathway. Our findings offer some novel insights into the RNA editing function in the mammary gland of yaks.

**Abstract:**

The mammary gland is a complicated organ comprising several types of cells, and it undergoes extensive morphogenetic and metabolic changes during the female reproductive cycle. RNA editing is a posttranscriptional modification event occurring at the RNA nucleotide level, and it drives transcriptomic and proteomic diversities, with potential functional consequences. RNA editing in the mammary gland of yaks, however, remains poorly understood. Here, we used REDItools to identify RNA editing sites in mammary gland tissues in yaks during the lactation period (LP, *n* = 2) and dry period (DP, *n* = 3). Totally, 82,872 unique RNA editing sites were identified, most of which were detected in the noncoding regions with a low editing degree. In the coding regions (CDS), we detected 5235 editing sites, among which 1884 caused nonsynonymous amino acid changes. Of these RNA editing sites, 486 were found to generate novel possible miRNA target sites or interfere with the initial miRNA binding sites, indicating that RNA editing was related to gene regulation mediated by miRNA. A total of 14,159 RNA editing sites (involving 3238 common genes) showed a significant differential editing level in the LP when compared with that in the DP through Tukey’s Honest Significant Difference method (*p* < 0.05). According to the Kyoto Encyclopedia of Genes and Genomes (KEGG) analysis, genes that showed different RNA editing levels mainly participated in pathways highly related to mammary gland development, including MAPK, PI3K-Akt, FoxO, and GnRH signaling pathways. Collectively, this work demonstrated for the first time the dynamic RNA editome profiles in the mammary gland of yaks and shed more light on the mechanism that regulates lactation together with mammary gland development.

## 1. Introduction

RNA editing refers to a newly discovered mechanism that alters the RNA sequence itself without altering its genomic DNA (gDNA) through nucleotide deletion, insertion, and substitution [1]. These types of modifications may occur within the coding or noncoding regions, which result in transcriptome plasticity and diversity along with changing amino acid, impacting alternative splicing (AS), affecting RNA stability, and modulating the nuclear retention of RNAs [2,3]. There are two major RNA editing types in mammals, including adenosine-to-inosine (A-to-I) editing (most frequently seen in vertebrates) under the catalysis of adenosine deaminase acting on RNA (ADAR) proteins [4] and C-to-U RNA editing under the catalysis of cytidine deaminases belonging to the apolipoprotein B-editing catalytic polypeptide-like (APOBEC) family [5]. The advancement of computational methods has enabled elucidating many RNA editing sites in human and mouse [6]. More recently, a few studies have focused on the function of RNA editing in farm animals, such as cattle [7,8], pig [9,10], sheep [11], goat [12], and chicken [13,14]. 

Yak (*Bos grunniens*), a multipurpose livestock, is widely distributed in the Qinghai-Tibetan plateau and adjacent regions [15]. It not only provides meat, but also milk and wool. Yak milk is known as concentrated milk because of its superiority in terms of nutrient contents such as proteins, bioactive fatty acids, specific enzymes, antioxidant vitamins, and probiotic bacteria [16,17]. Yak milk and its corresponding milk products are an important component of the daily diet for local Tibetan residents, and they exert an important influence in maintaining the health of Tibetans following hypoxia. Given the significance of yak milk in high-altitude regions, several researchers are now focusing their attention on enhancing milk quality and yield.

The mammary gland is an endocrine organ related to milk production and delivery, and its function and physiology are substantially altered in pregnancy [18]. Next-generation sequencing (NGS) technique has enabled the identification of genes and noncoding RNAs related to lactogenesis, which provides new opportunities to discover the hidden mechanisms of milk synthesis and mammary gland development in yaks [19,20,21]. There is, however, no available information on how RNA editing sites affect the lactation regulation and mammary gland development of yaks. In the present study, we systematically characterized RNA editing profiles by whole-genome resequencing and strand-specific RNA sequencing (RNA-seq) information for the yak mammary gland in the lactation period (LP) and dry period (DP). This study also examined the potential functions of such RNA editing events in mammary gland development and offered novel insights in improving yak milk yield and quality.

## 2. Materials and Methods

### 2.1. Description of Datasets

For identifying RNA editing profiles in the yak mammary gland at the LP and DP, we retrieved five paired-end RNA-seq datasets from our prior work [21]. Total RNA was extracted using TRIzol reagent (Invitrogen, Waltham, MA, USA) according to the manufacturer’s protocol. After assessing the RNA quality, 3 µg of total RNA from each sample was used for RNA-seq library preparation. The libraries were developed from five female Ashidan yaks with no mastitis. Of these yaks, two were in the LP, and the remaining three were in the DP. Libraries were sequenced on the Illumina HiSeq 2500 platform. The sequencing data are available on the NCBI BioProject under accession number PRJNA626061.

### 2.2. Whole-Genome Sequencing 

We extracted gDNA from the yak blood specimen that had the same genetic background as the RNA-seq samples. All the animal experiments were performed in accordance with the guidelines of the Animal Administration and Ethics Committee of Lanzhou Institute of Husbandry and Pharmaceutical Sciences of CAAS (Approval No. SYXK-2014-0002). The MGIEasy Universal DNA Library Prep Kit (MGI, Shenzhen, China) was adopted for constructing DNA libraries in accordance with the BGI’s standard preparation protocol. We sequenced the library as 150 base-pair (bp) paired-end runs by using MGISEQ2000 (MGI, Shenzhen, China) at Frasergen Bioinformatics Co., Ltd. (Wuhan, China).

### 2.3. Read Mapping 

After removing ploy-N, low-quality bases, and contaminating adapter molecules from the raw data, we aligned DNA clean reads to the domestic yak genome sequence (LU_ Bosgru_v3.0) by BWA (v0.7.17) [22]. Samtools 1.3 [23] was used to sort mapped reads, and duplicate reads were eliminated MarkDuplicate (Picard tools 2.13.2, https://broadinstitute.github.io/picard, (accessed on 30 October 2017). Additionally, we used Hisat2 (v2.1.0) [24] to map RNA-seq clean reads into reference genomes by adopting the default parameters. Samtools 1.3 [23] was used to sort the reads and convert them to the BAM format.

### 2.4. Detection and Annotation of RNA Editing Sites

The REDItoolDnaRna.py script (REDItools) [25] was used to detect RNA editing events. To ensure the accurate identification of editing sites, we used the below-mentioned parameters for limiting false positives: -c 10 (minimum read coverage, 10), -m 25 (minimum mapping quality score, 25), -q 25 (minimum quality score, 25), -O 5 (minimum homopolymeric length, 5), -n 0.05 (minimum editing frequency, 0.05), and -v 3 (minimum number of reads supporting the variation, 3) [26]. Finally, we retained the edited sites that were identified in at least two of the five individuals. SnpEff version 4.3 [27] was used to annotate the genomic feature of RNA editing sites. During annotation analysis, the SnpEff database was constructed using the domestic yak reference genome (LU_ Bosgru_v3.0) and corresponding genome annotation GFF3 file.

### 2.5. Conservation Analysis of RNA Editing Sites

For conservation analysis, we aligned the 25-bp sequence in upstream and downstream of candidate RNA editing sites against 50-bp flanking regions of the reported human sites by employing the NCBI BLAST tools blastn. We deemed alignments with e-values < 1 × 10^−5^ and identity more than 85% to be the conserved editing sites. This study obtained known human RNA editing sites based on REDIportal (http://srv00.recas.ba.infn.it/atlas/, (accessed on 23 March 2021) databases [28]. We used a total of 4,627,557 human A-to-I editing sites.

### 2.6. Analysis of the RNA Editing Effects on miRNA Regulation

Regarding the editing sites in mRNA 3′ untranslated regions (UTRs), we adopted RNAhybrid (-b 1 -c -f 2,8 -m 100,000 -u 1 -v 1 -e -10) [29] and miRanda (sc 140 -en -10 -scale 4 -strict) [30] for predicting miRNA target sites in edited and unedited sequences. Interaction of miRNA with mRNA existing in the edited type sequences but not in the unedited type sequence was considered as interaction gain. In contrast, the miRNA–mRNA interaction that existed in the unedited type sequence but not in the edited type sequence was considered as interaction loss.

### 2.7. Pairwise Comparison of RNA Editing Sites between the Groups

In order to illustrate RNA editing sites related to lactation and mammary gland growth at the genome-wide scale, we used Tukey’s Honest Significant Difference method to identify RNA-edited sites as significantly different (*p* < 0.05) between the LP and DP. 

### 2.8. Functional Analysis

G:Profile web tool [31] was used for Gene Ontology (GO) analysis. In addition, KOBAS 3.0 software [32] was used for Kyoto Encyclopedia of Genes and Genomes (KEGG) analysis through the hypergeometric test. We then deemed pathways with FDR < 0.05 to be significantly enriched. 

## 3. Results

### 3.1. Identification of RNA Editing Sites in the Mammary Gland

**Figure 1:**
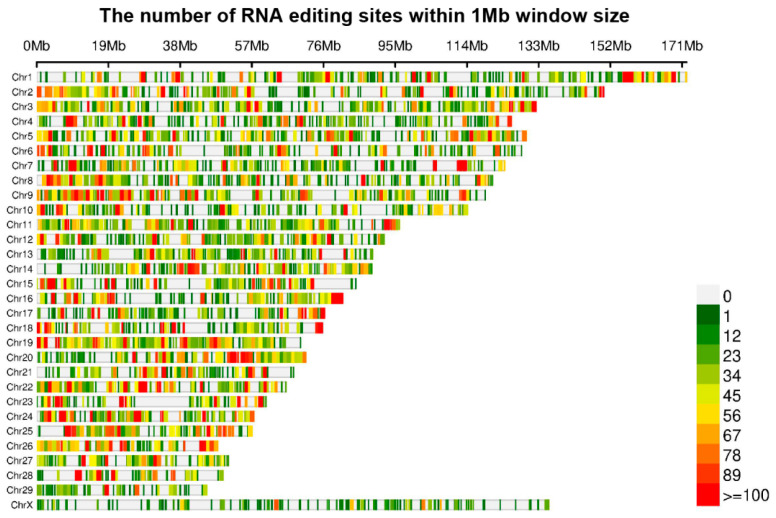
Distribution of putative RNA editing sites across yak chromosomes.

**Figure 2:**
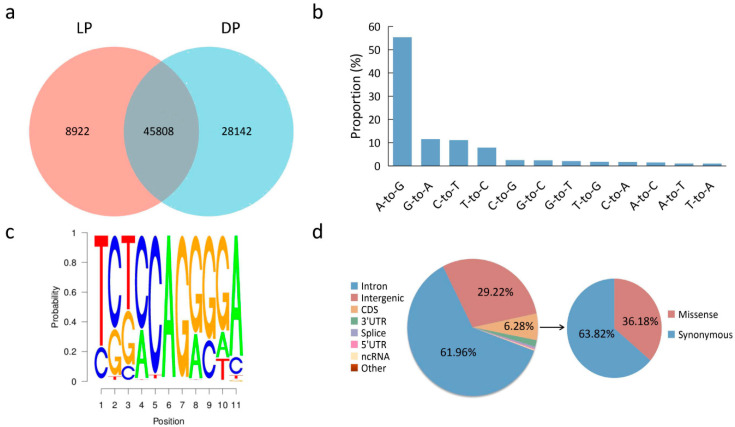
Signatures of the editome in the mammary gland. (**a**) Number of shared editing sites between LP and DP. (**b**) Distribution of RNA editing types. (**c**) A-to-G RNA editing motif. (**d**) Distribution of the identified RNA editing sites across different genomic locations.

To detect genome-wide RNA editing sites in the yak mammary gland, a set of strand-specific RNA-seq data from five female yaks was obtained from our previous study. After filtering low-quality reads and adapter sequences, more than 20 GB of raw bases were obtained in each sample. By applying a strict filter for excluding false positives, we discovered a total of 82,872 specific RNA editing sites among the samples (Table S1). A wide and uniform distribution of the identified RNA editing sites was observed among yak chromosomes, with chromosome 1 containing the most number of RNA editing sites, followed by chromosome 9 and chromosome 3 (Figure 1). The number of RNA editing sites varied between the LP and DP. Totally, 45,808 RNA editing sites were shared by the LP and DP, 8922 RNA editing sites were found only in the LP, and 28,142 RNA editing sites were found only in the DP (Figure 2a). 

### 3.2. Characterization of RNA Editing Sites in the Mammary Gland

Totally, 12 RNA editing types were detected, which included A-to-G, A-to-C, A-to-T, C-to-A, C-to-G, C-to-T, G-to-A, G-to-C, G-to-T, T-to-A, T-to-C, and T-to-G (Figure 2). Among all the editing types, 66.51% were of the canonical type (A-to-I and C-to-T) (Figure 2b). We further checked flanking sequences of the A-to-G editing sites and determined the sequence preference of the A-to-G editing site. Our results showed that the one nucleotide upstream of the editing site presented depleted G, while the one nucleotide downstream showed enriched G (Figure 2c). These findings were consistent with the known mammalian ADAR substrates for preferred target sequences [33,34].

### 3.3. Distribution of RNA Editing Sites across Different Genomic Regions 

As shown in Figure 2d, the number of RNA editing sites varied among the different genomic regions. Most sites were detected within introns (61.96%), followed by intergenic region (29.22%). Of these, the RNA editing sites located in the intergenic region were near annotated genes, and some sites may be extended 5′ UTRs or 3′ UTRs. Moreover, we detected 5235 (6.32%) editing sites in the coding regions (CDS), while 63.82% and 36.18% editing sites were synonymous and nonsynonymous variants, respectively. Among the detected editing sites, 58,441 editing sites were located within the 6622 annotated genes and were used for further analysis (Table S2).

### 3.4. Effects of RNA Editing Sites on miRNA–mRNA Interactions

**Figure 3:**
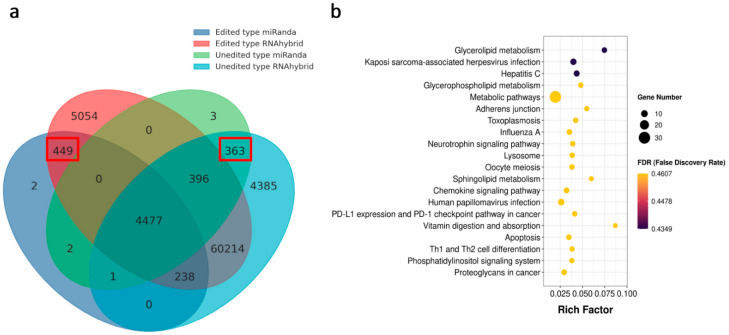
The influence of RNA editing sites on miRNA regulation. (**a**) Overall statistics of the RNA editing sites that altered miRNA binding capacity. The altered interactions are marked with red boxes. (**b**) KEGG enrichment analysis of genes with modified miRNA binding sites. Rich Factor is the ratio of differentially edited gene numbers annotated in this pathway term to all gene numbers annotated in this pathway term.

Similar to miRNA binding site single nucleotide polymorphisms (SNPs), RNA editing sites within miRNA binding sites may influence the recognition between miRNA and target mRNA. Around 1.63% of the RNA editing sites were located within 3′ UTR, which included 486 sites that were predicted to change miRNA binding capacity. Among these 486 RNA editing sites, 294 produced 449 novel possible miRNA binding sites, and 237 destroyed 363 miRNA target sites (Table S3). This study discovered 348 affected target genes (Figure 3a). To analyze the functions of the impacted target genes, we conducted GO and KEGG analyses. In the GO analysis, the affected target genes were mainly enriched in 13 terms (Table S4). According to KEGG pathway analysis, the impacted target genes were mainly involved in glycerolipid metabolism, metabolic pathways, adherens junction, sphingolipid metabolism, and apoptosis (Figure 3b).

### 3.5. Cross-Species Analysis between Yak and Human

To examine the conservation of RNA editing sites in human and yak, this study performed cross-species comparison by conducting BLAST analysis. When strict thresholds were applied to the results (identity >85%, e-value < 1 × 10^−5^), 74 conserved RNA editing sites were identified in 36 genes (Table S5). Some of the edited genes of yaks are homologous to human genes, such as SON DNA binding protein (*SON*), forkhead box O3 (*FOXO3*), ArfGAP With RhoGAP domain, ankyrin repeat and PH domain 1 (*ARAP1*), Nei-like DNA glycosylase 1 (*NEIL1*), THO complex 1 (*THOC1*), H2A clustered histone 20 (*H2AC20*), chromatin target Of PRMT1 (*CHTOP*), cyclin-dependent kinase 13 (*CDK13*), and insulin-like growth factor binding protein 7 (*IGFBP7*). Of these, editing sites in *SON*, *NEIL1*, *CDK13,* and *IGFBP7* genes may lead to missense mutations. 

### 3.6. Distribution of RNA Editing Sites among Different Physiological Stages

**Figure 4:**
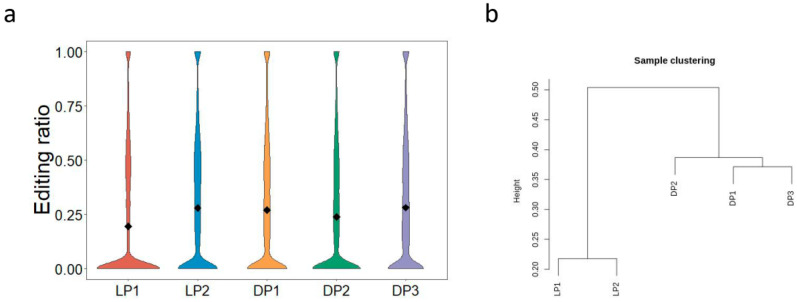
Features of RNA editing levels within and between groups. (**a**) The violin plot of editing levels across samples. (**b**) Hierarchical clustering of RNA editing levels across five samples.

**Figure 5:**
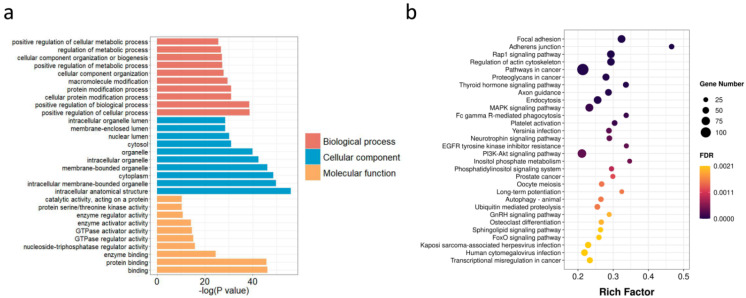
GO and KEGG enrichment of genes with differential editing levels. (**a**) Top30 GO enrichment terms. (**b**) Top30 KEGG pathway enrichment terms.

Surprisingly, the number of RNA editing sites was obviously different among diverse samples, but the average editing level of detected sites was similar (mean level, 0.19–0.28) (Figure 4a). On the basis of hierarchical clustering, fewer heterogeneities in editing levels were detected in intra-group than in inter-group (Figure 4b), indicating that the transcriptome-wide RNA editing level could be used to characterize the physiological changes in the mammary gland. We identified differentially edited sites between the LP and DP by using Tukey’s Honest Significant Difference method. A total of 14,159 sites in 3238 genes was differentially edited between the LP and DP (*p* < 0.05) (Table S6). The GO analysis showed that genes with differential editing levels were remarkably enriched into 1256 terms (Table S7). In the biological process (BP) category, the most significantly enriched terms were positive regulation of cellular process, positive regulation of biological process, and cellular protein modification process. In the molecular function (MF) category, the most significantly enriched terms were protein binding, enzyme binding, and nucleoside-triphosphatase regulator activity. In the cellular component (CC) category, intracellular anatomical structure, intracellular membrane-bounded organelle, and cytoplasm were the most significantly enriched terms (Figure 5a). As suggested by the KEGG analysis, genes with differential editing levels were enriched in 124 pathways, including focal adhesion pathway, MAPK signaling pathway, PI3K-Akt signaling pathway, GnRH signaling pathway, and FoxO signaling pathway (Figure 5b; Table S8).

## 4. Discussion

RNA editing is an important source of molecular diversity that increases transcript flexibility and diversity by altering specific nucleotides within RNA posttranscriptionally. With the rapid development and application of NGS technologies, numerous RNA editing sites have been characterized in the genome of diverse animal species. To date, the role of RNA editing in the development of the mammary gland is ambiguous. In the present study, we comprehensively identified the genome-wide RNA editing events in the yak mammary gland.

In this study, we identified 82,872 editing sites in yak mammary tissues at the LP and DP. Notably, the number of editing sites was significantly different between the LP and DP. Compared to the LP, a higher number of editing sites was identified in the DP, which indicated higher RNA editing activity. Twelve types of RNA editing were detected, among which the proportion of A-to-G was the highest. Among all the RNA editing types, A-to-G and C-to-T were identified to be the canonical RNA editing events, whereas the remaining RNA editing types were the non-canonical ones [35]. The A-to-G RNA editing event in double-stranded RNA (dsRNA), mediated by the ADAR (adenosine deaminase acting on RNA) family of enzymes, is regarded as the most common type of frequent editing event in mammals [4]. Additionally, a high proportion of the A-to-G editing type suggests the high accuracy of our results.

Consistent with previous studies on cattle and pig [7,10], our data revealed that the majority of RNA editing sites were located in the intron and intergenic region, suggesting RNA editing may modulate alternative splicing and nuclear retention. Moreover, intron and intergenic RNA editing sites are also assumed to influence the secondary structures of long noncoding RNA (lncRNA) [36]. As lncRNAs from the yak reference genome are incompletely annotated, the present study did not investigate how RNA editing sites affected the secondary structures of lncRNAs; this is our future research direction. 

Annotation of the edited genes with missense mutations in the mammary gland revealed that some of the genes were associated with mammary gland development and milk traits. For instance, BRCA1 DNA repair associated (*BRCA1*), a breast cancer susceptibility gene, is involved in lobular-alveolar development in the mammary gland [37,38]. Lactotransferrin (*LTF*) encodes a major iron-binding protein in milk, which has both bacteriostatic and bactericidal activity [39]. Polymorphism in this gene is associated with milk performance traits of Holstein cattle [40]. Nuclear receptor-interacting protein 1 (*NRIP1*), also known as *RIP140*, is a co-regulator for transcription factors that regulate mouse mammary gland development [41]. Therefore, these missense editing sites may play important roles in yak lactation and mammary gland development. In addition to missense editing sites, many RNA editing sites detected in the present study were discovered in 3′ UTRs in diverse transcripts, which can modulate mRNA availability and translation efficiency by creating novel potential miRNA binding sites and disrupting the existing miRNA binding sites. According to the GO and KEGG analysis, such target genes were enriched in some critical pathways associated with lactation biology, such as glycerolipid metabolism [42], sphingolipid metabolism [43], and apoptosis [44]. Based on these results, RNA editing sites within 3′ UTRs exert *cis*-regulatory impact on gene expression by influencing mRNA degradation and stabilization.

In this study, we found 74 conserved sites in 36 genes, which were reported in human RNA from REDIportal databases. Similar to the previous result [10], the level of overlap between editing sites identified in this study and the reported human editing sites was low, which indicated that only a few RNA editing sites are conserved across large evolutionary distances. Some of the conserved edited genes of yaks are homologous to human genes, such as *SON*, *NEIL1*, *FOXO3*, *CDK13*, and *IGFBP7*. Although most of the functional alterations resulting from these editing sites are unknown, the conservation of these sites indicates that their functions are important among mammals.

Our genome-wide differential RNA editing analyses yielded a set of 14,159 sites that were significantly differentially edited between the LP and DP. The KEGG analysis showed that the genes with differential editing level were enriched in some critical pathways related to lactation and the mammary gland, which included focal adhesion pathway, MAPK signaling pathway, PI3K-Akt signaling pathway, FoxO signaling pathway, GnRH signaling pathway, and ECM-receptor interaction pathway. Among these, the PI3K-Akt pathway exerts a key role during mammary gland development [45]. Insulin-like growth factor 1 (*IGF1*) is an amplification factor of the PI3K/AKT pathway, which is considered the regulatory factor for mammary gland growth. *IGF1* overexpression has been previously shown in pregnant or lactating mice, which leads to delayed mammary epithelial cell apoptosis in the declining phase of lactation [46]. Furthermore, in Polish Holstein Friesian cows, polymorphism of the *IGF1* gene is associated with milk fat and protein yields [47]. The MAPK signaling pathway is involved in mammary branching morphogenesis, which has influence on mammary gland development [48]. Do et al. investigated the expression profiles of miRNAs in bovine milk fat during the entire lactation curve, and the results revealed that some target genes of differentially expressed miRNAs were significantly enriched in the MAPK signaling pathway [49]. Wang et al. found the MAPK signaling pathway was enriched by target genes of up-regulated miRNAs in the non-lactating mammary gland of ewes [50]. Additionally, target genes of exosomal miRNAs in pigeon milk were found to involve the MAPK signaling pathway [51]. Interestingly, Mitogen-activated protein kinase 1 (MAPK1) was differentially edited between the LP and DP. This gene belongs to the MAPK signaling pathway, which was reported to affect the production of milk protein in mammary epithelial cells of dairy cows [52]. Insulin-induced gene 1 (*INSIG1*), an endoplasmic reticulum membrane protein, has an important function in regulating lipid synthesis [53]. In goat mammary epithelial cells, miR-26a and miR-26b regulate triacylglycerol accumulation and unsaturated fatty acid synthesis by binding to *INSIG1* [54]. Signal transducer and activator of transcription 3 (*STAT3*) belongs to the STAT protein family and participate in the mouse mammary epithelial cell apoptosis [55]. Co-expression network and pathway analysis revealed significant correlations between miR-18a and lactose content in cow’s milk [56]. It is reported that STAT3 might transcriptionally activate miR-18a and other members of miR-17/92 expression by binding to their promoter [57]. STAT3 is predicted to be a target of differentially expressed miRNAs in the mammary gland tissue of dairy goats at different developmental stages [58]. Fibroblast growth factor 10 (*FGF10*) regulates mammary gland development and homeostasis by acting through the fibroblast growth factor receptor 2B (*FGFR2B*) [59]. These observations suggest that RNA editing could be closely related to mammary development and lactation physiology. Although these RNA editing sites require further experimental validation, this information suggests that RNA editing is one of the potential mechanisms that could be used as the basis for quantitative locus (QTL) analysis of milk traits.

## 5. Conclusions

To the best of our knowledge, the present study is the first to detect RNA editing sites in the mammary gland of yaks. We comprehensively analyzed the landscape of RNA editing in the yak mammary gland in the LP and DP. On the basis of these findings, some genes containing the functional edited sites were found to be associated with mammary growth and lactation biology. Most importantly, these findings offer new insights into the mechanism underlying yak mammary gland development at the molecular level and enlarge the yak RNA editing site list.

## Data Availability

Transcriptomic data (SRA; PRJNA626061) from our previous study were used in in this study. The detailed information was described in the main text.

## Supplementary Materials

The following supporting information can be downloaded at: https://www.mdpi.com/article/10.3390/ani12020207/s1. Table S1: List of the identified RNA editing sites in mammary gland of yak. Table S2: Features of all the identified RNA editing sites. Table S3: List of RNA editing sites that altered miRNA binding capacity. Table S4: GO enrichment of genes with modified miRNA binding sites. Table S5: List of conserved RNA editing between yak and human. Table S6: List of differentially edited RNA editing sites between the LP and DP. Table S7: GO enrichment of genes with differential editing levels. Table S8: KEGG enrichment of genes with differential editing levels.
